# (*E*)-3-(Biphenyl-4-yl)-1-(3-bromo­phen­yl)prop-2-en-1-one

**DOI:** 10.1107/S1600536809043384

**Published:** 2009-10-28

**Authors:** Grzegorz Dutkiewicz, C. S. Chidan Kumar, H. S. Yathirajan, B. Narayana, Maciej Kubicki

**Affiliations:** a Department of Chemistry, Adam Mickiewicz University, Grunwaldzka 6, 60-780 Poznań, Poland; b Department of Studies in Chemistry, University of Mysore, Manasagangotri, Mysore 570 006, India; c Department of Studies in Chemistry, Mangalore University, Mangalagangotri 574 199, India

## Abstract

In the title compound, C_21_H_15_BrO, there are two planar rings connected through a conjugated double bond. As it crystallizes in a non-centrosymmetric space group it can be regarded as a good candidate for non-linear optical applications. The mol­ecule adopts an *E* configuration and the C—C=C—C torsion angle is 177.1 (4)°. The overall conformation of the compound may be described by the values of dihedral angles between the approximately planar parts. The terminal rings are twisted by an angle of 51.52 (9)°, while the biphenyl part is almost planar, the dihedral angle between the planes of the rings being 4.44 (17)°. The unit cell has one long dimension, above 35 Å, characteristic also of a majority of related compounds. The mol­ecules pack head-to-tail along this direction. C—H⋯π inter­actions are observed in the crystal structure.

## Related literature

For applications of chalcones, see: Cho *et al.* (1996 ▶); Dinkova-Kostova *et al.*, (1998 ▶); Fichou *et al.* (1988 ▶); Liu *et al.* (2003 ▶); Nielson *et al.* (1998 ▶); Rajas *et al.* (2002 ▶); Sarojini *et al.* (2006 ▶). For related structures, see: Fischer *et al.* (2007*a* ▶,*b* ▶,*c* ▶); Moorthi *et al.* (2007 ▶); Sarojini *et al.* (2007 ▶).
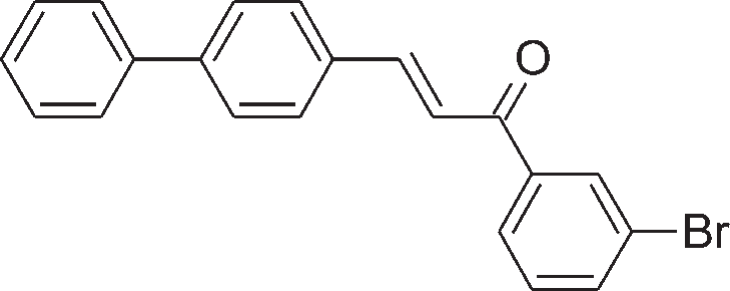


## Experimental

### 

#### Crystal data

C_21_H_15_BrO
*M* *_r_* = 363.24
Orthorhombic, 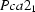

*a* = 6.092 (1) Å
*b* = 7.295 (1) Å
*c* = 36.619 (2) Å
*V* = 1627.4 (4) Å^3^
*Z* = 4
Mo *K*α radiation
μ = 2.53 mm^−1^
*T* = 295 K
0.4 × 0.2 × 0.2 mm

#### Data collection

Oxford Diffraction Xcalibur Sapphire2 (large Be window) diffractometer
Absorption correction: multi-scan (*CrysAlis Pro*; Oxford Diffraction, 2006 ▶) *T*_min_ = 0.632, *T*_max_ = 1.000
5236 measured reflections
2766 independent reflections
2209 reflections with *I* > 2σ(*I*)
*R*_int_ = 0.022

#### Refinement

*R*[*F* ^2^ > 2σ(*F* ^2^)] = 0.035
*wR*(*F* ^2^) = 0.085
*S* = 1.05
2766 reflections
208 parameters
1 restraint
H-atom parameters constrained
Δρ_max_ = 0.25 e Å^−3^
Δρ_min_ = −0.45 e Å^−3^
Absolute structure: Flack (1983 ▶), 1133 Friedel pairs
Flack parameter: 0.059 (11)

### 

Data collection: *CrysAlis Pro* (Oxford Diffraction, 2006 ▶); cell refinement: *CrysAlis Pro*; data reduction: *CrysAlis Pro*; program(s) used to solve structure: *SIR92* (Altomare *et al.*, 1994 ▶); program(s) used to refine structure: *SHELXL97* (Sheldrick, 2008 ▶); molecular graphics: *Stereochemical Workstation Operation Manual* (Siemens, 1989 ▶); software used to prepare material for publication: *SHELXL97*.

## Supplementary Material

Crystal structure: contains datablocks I, global. DOI: 10.1107/S1600536809043384/nk2009sup1.cif

Structure factors: contains datablocks I. DOI: 10.1107/S1600536809043384/nk2009Isup2.hkl

Additional supplementary materials:  crystallographic information; 3D view; checkCIF report

## Figures and Tables

**Table 1:** Hydrogen-bond geometry (Å, °)

*D*—H⋯*A*	*D*—H	H⋯*A*	*D*⋯*A*	*D*—H⋯*A*
C3—H3⋯*Cg*1^i^	0.93	2.85	3.583 (5)	137
C6—H6⋯*Cg*1^ii^	0.93	2.78	3.516 (5)	137
C9—H9⋯*Cg*2^i^	0.93	2.87	3.544 (5)	131
C12—H12⋯*Cg*2^ii^	0.93	2.97	3.655 (5)	131
C21—H21⋯*Cg*3^iii^	0.93	2.83	3.505 (5)	131

Symmetry codes: (i) 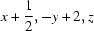; (ii) 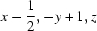; (iii) 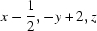. *Cg*1, *Cg*2 and *Cg*3 are the centroids of the C1–C6, C7–C12 and C17–C22 rings, respectively.

**Fig. 1.**
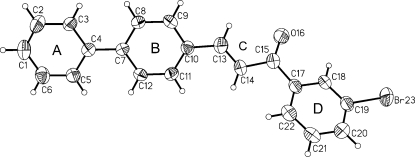
Anisotropic ellipsoid representation of the compound I together with atom labelling scheme. The ellipsoids are drawn at 50% probability level, hydrogen atoms are depicted as spheres with arbitrary radii.

**Fig. 2.**
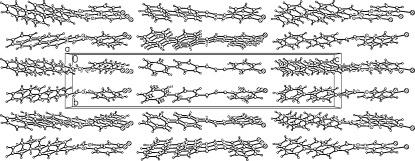
The crystal packing as seen along [100] direction.

## supplementary crystallographic information

### Comment

For such a structurally simple group of compounds, chalcones have displayed an impressive array of biological activities, among which antimalarial (Liu *et al.*, 2003), antiprotozoal (Nielson *et al.*, 1998), nitric oxide inhibition (Rajas *et al.*, 2002) and anticancer (Dinkova-Kostova *et al.*, 1998) activities have been cited in the literature. Also, among organic compounds reported for non-linear optical (NLO) properties, chalcone derivatives are notable materials for their excellent blue-light transmittance and good crystallizability. They provide the necessary configuration to show NLO properties, with two planar rings connected through a conjugated double bond (*e.g.*, Sarojini *et al.*, 2006). Substitution on either of the benzene rings greatly influences the non-centrosymmetric crystal packing. It is speculated that, in order to improve the activity, more bulky substituents should be introduced to increase the spontaneous polarization of non-centrosymmetric crystals (Fichou *et al.*, 1988). The molecular hyperpolarizability is strongly influenced, not only by the electronic effect, but also by the steric effect of the substituent (Cho *et al.*, 1996). Prompted by this, and in a continuation of our quest to synthesize new materials which can find use in the photonics industry, we have synthesized new chalcones and studied their SHG (second harmonic generation) efficiency.

(2*E*)-3-(biphenyl-4-yl)-1-(3-bromophenyl)prop-2-en-1-one (I) crystallizes in the non-centrosymmetric space group *Pca*2_1_, which makes NLO activity possible. The overall conformation of the molecule can be described by the dihedral angles between the planar fragments: two rings of biphenyl system (A and B, *cf.* Fig. 1), the enone fragment (C) and the (bromo)phenyl ring (D). All these fragments are in a good approximation planar (maximum deviation from the least-squares plane is 0.018 (4)Å for the enone fragment). The biphenyl rings are almost coplanar, the dihedral angle between them is 4.44 (17)°; the enone fragment is significantly inclined with respect to both neighbouring rings, B/C angle is 30.74 (11)° and C/D - 16.34 (12)°.

The conformation for the ketone system is *s–cis*, as evidenced by the torsion angle O16—C15— C14—C13 of -21.7 (6)°. In general, the conformation of the molecule (I) is similar to the related compounds (*e.g.*, Fischer *et al.*, 2007*a*, b, c, Moorthi *et al.*, 2007).

The unit cell of (I) has a long *c* axis of 36.619 (2) Å, and the molecules pack head-to-tail along this direction (Fig. 2). Such a long unit-cell parameter is observed in a number of similar compounds, even though they crystallize in different space groups and even in different crystal classes. For instance, 4-bromo (Fischer *et al.*, 2007*b*), 4-chloro (Fischer *et al.*, 2007*a*) and 4-methoxyphenyl (Fischer *et al.*, 2007*c*) analogues crystallize all in the *Cc* space groups with the long parameter (*ca*. 36 Å) along c-direction, 4-fluoro derivative (Sarojini *et al.*, 2007) - in *P*2_1_ space group (*Z*' = 2) with the long b direction *etc*. It might be also noted, that other unit-cell parameters in all these structures are also similar to those observed in (I), and the comparison of the packing modes shows a significant degree of isostructurality. This suggests that the same interactions are responsible for the crystal packing in these structures: these can be some relatively short and linear C—H···π contacts, and van der Waals interactions.

### Experimental

5 ml 40% KOH solution was added to a thoroughly stirred solution of 3-bromoacetophenone (1.0 g, 5 m mol) and 4-biphenylcarboxaldehyde (1.0 g, 5.4 m mol) in 15 ml of methanol. The mixture was stirred overnight and filtered. The product formed was crystallized in methanol. X-ray quality crystals were grown from slow evaporation of ethyl acetate solution (m.p.: 378 – 380 K).

### Refinement

Hydrogen atoms were placed in idealized positions, and refined as riding. Their isotropic thermal parameters were set at 1.2 times *U*_eq_'s of appropriate carrier atoms.

### Crystal data

**Table:**

C_21_H_15_BrO	*F*(000) = 736
*M**_r_* = 363.24	*D*_x_ = 1.483 Mg m^−^^3^
Orthorhombic, *P**c**a*2_1_	Mo *K*α radiation, λ = 0.71073 Å
Hall symbol: P 2c -2ac	Cell parameters from 2900 reflections
*a* = 6.092 (1) Å	θ = 2.2–26.8°
*b* = 7.295 (1) Å	µ = 2.53 mm^−^^1^
*c* = 36.619 (2) Å	*T* = 295 K
*V* = 1627.4 (4) Å^3^	Prism, colourless
*Z* = 4	0.4 × 0.2 × 0.2 mm

### Data collection

**Table:**

Oxford Diffraction Xcalibur Sapphire2 (large Be window) diffractometer	2766 independent reflections
Radiation source: Enhance (Mo) X-ray Source	2209 reflections with *I* > 2σ(*I*)
graphite	*R*_int_ = 0.022
Detector resolution: 8.1929 pixels mm^-1^	θ_max_ = 26.9°, θ_min_ = 2.2°
ω scans	*h* = −5→7
Absorption correction: multi-scan (*CrysAlis PRO*; Oxford Diffraction, 2006)	*k* = −5→9
*T*_min_ = 0.632, *T*_max_ = 1.000	*l* = −45→41
5236 measured reflections	

### Refinement

**Table:**

Refinement on *F*^2^	Secondary atom site location: difference Fourier map
Least-squares matrix: full	Hydrogen site location: inferred from neighbouring sites
*R*[*F*^2^ > 2σ(*F*^2^)] = 0.035	H-atom parameters constrained
*wR*(*F*^2^) = 0.085	*w* = 1/[σ^2^(*F*_o_^2^) + (0.050*P*)^2^] where *P* = (*F*_o_^2^ + 2*F*_c_^2^)/3
*S* = 1.05	(Δ/σ)_max_ = 0.001
2766 reflections	Δρ_max_ = 0.25 e Å^−^^3^
208 parameters	Δρ_min_ = −0.45 e Å^−^^3^
1 restraint	Absolute structure: Flack (1983), 1133 Friedel pairs
Primary atom site location: structure-invariant direct methods	Flack parameter: 0.059 (11)

### Special details

**Table:**

Geometry. All e.s.d.'s (except the e.s.d. in the dihedral angle between two l.s. planes) are estimated using the full covariance matrix. The cell e.s.d.'s are taken into account individually in the estimation of e.s.d.'s in distances, angles and torsion angles; correlations between e.s.d.'s in cell parameters are only used when they are defined by crystal symmetry. An approximate (isotropic) treatment of cell e.s.d.'s is used for estimating e.s.d.'s involving l.s. planes.
Refinement. Refinement of *F*^2^ against ALL reflections. The weighted *R*-factor *wR* and goodness of fit *S* are based on *F*^2^, conventional *R*-factors *R* are based on *F*, with *F* set to zero for negative *F*^2^. The threshold expression of *F*^2^ > σ(*F*^2^) is used only for calculating *R*-factors(gt) *etc*. and is not relevant to the choice of reflections for refinement. *R*-factors based on *F*^2^ are statistically about twice as large as those based on *F*, and *R*- factors based on ALL data will be even larger.

### Fractional atomic coordinates and isotropic or equivalent isotropic displacement parameters (Å^2^)

**Table:**

	*x*	*y*	*z*	*U*_iso_*/*U*_eq_	
C1	0.6509 (7)	0.7425 (7)	0.79823 (11)	0.0516 (11)	
H1	0.5927	0.7379	0.7748	0.062*	
C2	0.8449 (7)	0.8289 (5)	0.80447 (11)	0.0532 (10)	
H2	0.9194	0.8847	0.7853	0.064*	
C3	0.9320 (7)	0.8337 (5)	0.83967 (9)	0.0430 (9)	
H3	1.0645	0.8938	0.8437	0.052*	
C4	0.8253 (6)	0.7506 (5)	0.86911 (10)	0.0333 (7)	
C5	0.6238 (6)	0.6661 (4)	0.86149 (11)	0.0407 (9)	
H5	0.5454	0.6120	0.8804	0.049*	
C6	0.5403 (7)	0.6612 (5)	0.82712 (12)	0.0504 (10)	
H6	0.4072	0.6026	0.8229	0.061*	
C7	0.9218 (6)	0.7517 (5)	0.90615 (9)	0.0302 (7)	
C8	1.1289 (5)	0.8263 (5)	0.91272 (10)	0.0376 (8)	
H8	1.2081	0.8759	0.8934	0.045*	
C9	1.2194 (6)	0.8281 (5)	0.94747 (11)	0.0390 (8)	
H9	1.3586	0.8776	0.9508	0.047*	
C10	1.1075 (6)	0.7580 (5)	0.97729 (10)	0.0364 (8)	
C11	0.9024 (6)	0.6836 (5)	0.97075 (10)	0.0424 (9)	
H11	0.8234	0.6346	0.9902	0.051*	
C12	0.8120 (6)	0.6801 (5)	0.93638 (11)	0.0398 (8)	
H12	0.6738	0.6284	0.9332	0.048*	
C13	1.2154 (6)	0.7575 (6)	1.01312 (11)	0.0449 (9)	
H13	1.3613	0.7952	1.0136	0.054*	
C14	1.1287 (7)	0.7096 (6)	1.04483 (11)	0.0494 (10)	
H14	0.9815	0.6761	1.0461	0.059*	
C15	1.2648 (7)	0.7089 (5)	1.07855 (11)	0.0476 (9)	
O16	1.4619 (5)	0.6988 (4)	1.07694 (8)	0.0696 (9)	
C17	1.1481 (6)	0.7302 (5)	1.11410 (10)	0.0412 (8)	
C18	1.2662 (6)	0.6895 (4)	1.14615 (10)	0.0380 (8)	
H18	1.4069	0.6405	1.1447	0.046*	
C19	1.1730 (6)	0.7225 (5)	1.17930 (11)	0.0435 (8)	
C20	0.9649 (8)	0.7894 (5)	1.18260 (12)	0.0515 (10)	
H20	0.9028	0.8092	1.2055	0.062*	
C21	0.8486 (6)	0.8273 (5)	1.15093 (14)	0.0487 (11)	
H21	0.7074	0.8751	1.1526	0.058*	
C22	0.9371 (7)	0.7956 (5)	1.11731 (11)	0.0477 (9)	
H22	0.8545	0.8183	1.0964	0.057*	
Br23	1.33920 (7)	0.67882 (6)	1.222156 (16)	0.06635 (16)	

### Atomic displacement parameters (Å^2^)

**Table:**

	*U*^11^	*U*^22^	*U*^33^	*U*^12^	*U*^13^	*U*^23^
C1	0.071 (3)	0.044 (2)	0.040 (2)	0.008 (2)	−0.014 (2)	−0.0040 (19)
C2	0.071 (3)	0.054 (2)	0.035 (2)	−0.006 (2)	0.0032 (17)	0.0005 (16)
C3	0.042 (2)	0.054 (2)	0.0327 (18)	−0.0073 (18)	0.0040 (14)	−0.0016 (15)
C4	0.038 (2)	0.0254 (16)	0.0363 (17)	0.0042 (15)	0.0029 (14)	−0.0008 (13)
C5	0.044 (2)	0.0343 (19)	0.044 (2)	−0.0048 (15)	−0.0018 (15)	0.0043 (15)
C6	0.057 (3)	0.045 (2)	0.049 (2)	−0.0094 (18)	−0.0123 (19)	0.0004 (18)
C7	0.0334 (19)	0.0240 (15)	0.0332 (16)	0.0007 (14)	0.0039 (13)	0.0004 (12)
C8	0.036 (2)	0.040 (2)	0.0362 (18)	−0.0046 (15)	0.0024 (13)	0.0039 (14)
C9	0.0300 (19)	0.041 (2)	0.046 (2)	−0.0043 (15)	−0.0044 (15)	−0.0022 (15)
C10	0.037 (2)	0.0369 (18)	0.0354 (18)	−0.0008 (15)	−0.0013 (13)	−0.0026 (15)
C11	0.043 (2)	0.048 (2)	0.036 (2)	−0.0089 (17)	0.0034 (15)	0.0064 (16)
C12	0.031 (2)	0.048 (2)	0.0408 (18)	−0.0061 (17)	−0.0008 (13)	−0.0014 (15)
C13	0.050 (2)	0.042 (2)	0.043 (2)	0.0038 (18)	−0.0044 (18)	−0.0012 (17)
C14	0.043 (2)	0.066 (3)	0.038 (2)	−0.0047 (19)	−0.0095 (16)	−0.0006 (18)
C15	0.046 (2)	0.056 (2)	0.040 (2)	−0.0019 (19)	−0.0052 (17)	−0.0007 (17)
O16	0.0414 (18)	0.118 (3)	0.0490 (17)	0.0122 (17)	−0.0012 (13)	−0.0013 (16)
C17	0.037 (2)	0.046 (2)	0.0412 (19)	−0.0037 (17)	−0.0081 (14)	−0.0013 (16)
C18	0.0331 (19)	0.0402 (19)	0.0406 (19)	−0.0016 (15)	−0.0073 (14)	−0.0010 (15)
C19	0.041 (2)	0.048 (2)	0.042 (2)	−0.0057 (18)	−0.0078 (15)	−0.0001 (16)
C20	0.051 (3)	0.058 (2)	0.045 (2)	−0.001 (2)	0.0027 (17)	−0.0004 (18)
C21	0.035 (2)	0.051 (2)	0.060 (3)	−0.0022 (18)	−0.0076 (17)	0.000 (2)
C22	0.044 (2)	0.047 (2)	0.052 (2)	−0.0032 (18)	−0.0090 (18)	0.0020 (16)
Br23	0.0653 (3)	0.0941 (3)	0.03960 (19)	0.0043 (2)	−0.0142 (2)	0.0021 (3)

### Geometric parameters (Å, °)

**Table:**

C1—C2	1.358 (6)	C11—C12	1.374 (5)
C1—C6	1.387 (6)	C11—H11	0.9300
C1—H1	0.9300	C12—H12	0.9300
C2—C3	1.394 (5)	C13—C14	1.323 (6)
C2—H2	0.9300	C13—H13	0.9300
C3—C4	1.397 (5)	C14—C15	1.487 (5)
C3—H3	0.9300	C14—H14	0.9300
C4—C5	1.401 (5)	C15—O16	1.204 (5)
C4—C7	1.478 (5)	C15—C17	1.492 (6)
C5—C6	1.358 (5)	C17—C22	1.376 (6)
C5—H5	0.9300	C17—C18	1.408 (5)
C6—H6	0.9300	C18—C19	1.361 (5)
C7—C12	1.395 (5)	C18—H18	0.9300
C7—C8	1.395 (5)	C19—C20	1.364 (6)
C8—C9	1.387 (5)	C19—Br23	1.894 (4)
C8—H8	0.9300	C20—C21	1.387 (6)
C9—C10	1.385 (5)	C20—H20	0.9300
C9—H9	0.9300	C21—C22	1.364 (6)
C10—C11	1.383 (5)	C21—H21	0.9300
C10—C13	1.468 (5)	C22—H22	0.9300
			
C2—C1—C6	119.5 (4)	C10—C11—H11	119.1
C2—C1—H1	120.3	C11—C12—C7	121.9 (3)
C6—C1—H1	120.2	C11—C12—H12	119.1
C1—C2—C3	119.9 (4)	C7—C12—H12	119.1
C1—C2—H2	120.0	C14—C13—C10	127.3 (4)
C3—C2—H2	120.1	C14—C13—H13	116.3
C2—C3—C4	121.7 (4)	C10—C13—H13	116.3
C2—C3—H3	119.2	C13—C14—C15	120.4 (4)
C4—C3—H3	119.2	C13—C14—H14	119.8
C3—C4—C5	116.4 (3)	C15—C14—H14	119.8
C3—C4—C7	121.4 (3)	O16—C15—C14	121.0 (4)
C5—C4—C7	122.2 (3)	O16—C15—C17	121.6 (3)
C6—C5—C4	121.6 (4)	C14—C15—C17	117.3 (4)
C6—C5—H5	119.2	C22—C17—C18	118.6 (4)
C4—C5—H5	119.2	C22—C17—C15	123.8 (3)
C5—C6—C1	120.9 (4)	C18—C17—C15	117.5 (3)
C5—C6—H6	119.5	C19—C18—C17	119.5 (4)
C1—C6—H6	119.5	C19—C18—H18	120.2
C12—C7—C8	116.3 (3)	C17—C18—H18	120.2
C12—C7—C4	122.4 (3)	C18—C19—C20	122.0 (4)
C8—C7—C4	121.3 (3)	C18—C19—Br23	119.1 (3)
C9—C8—C7	121.4 (3)	C20—C19—Br23	118.9 (3)
C9—C8—H8	119.3	C19—C20—C21	118.2 (4)
C7—C8—H8	119.3	C19—C20—H20	120.9
C10—C9—C8	121.6 (3)	C21—C20—H20	120.9
C10—C9—H9	119.2	C22—C21—C20	121.2 (4)
C8—C9—H9	119.2	C22—C21—H21	119.4
C11—C10—C9	116.9 (3)	C20—C21—H21	119.4
C11—C10—C13	124.0 (3)	C21—C22—C17	120.4 (4)
C9—C10—C13	119.0 (3)	C21—C22—H22	119.8
C12—C11—C10	121.9 (3)	C17—C22—H22	119.8
C12—C11—H11	119.1		
			
C6—C1—C2—C3	0.6 (6)	C4—C7—C12—C11	−179.3 (3)
C1—C2—C3—C4	0.3 (6)	C11—C10—C13—C14	−9.0 (7)
C2—C3—C4—C5	−1.4 (5)	C9—C10—C13—C14	174.4 (4)
C2—C3—C4—C7	178.2 (4)	C10—C13—C14—C15	177.1 (4)
C3—C4—C5—C6	1.7 (5)	C13—C14—C15—O16	−21.7 (6)
C7—C4—C5—C6	−178.0 (3)	C13—C14—C15—C17	155.4 (4)
C4—C5—C6—C1	−0.9 (6)	O16—C15—C17—C22	160.0 (4)
C2—C1—C6—C5	−0.3 (6)	C14—C15—C17—C22	−17.1 (5)
C3—C4—C7—C12	175.8 (3)	O16—C15—C17—C18	−16.7 (6)
C5—C4—C7—C12	−4.6 (5)	C14—C15—C17—C18	166.3 (3)
C3—C4—C7—C8	−3.8 (5)	C22—C17—C18—C19	−2.6 (5)
C5—C4—C7—C8	175.8 (3)	C15—C17—C18—C19	174.2 (3)
C12—C7—C8—C9	0.1 (5)	C17—C18—C19—C20	1.9 (6)
C4—C7—C8—C9	179.7 (3)	C17—C18—C19—Br23	−176.8 (3)
C7—C8—C9—C10	−0.7 (5)	C18—C19—C20—C21	−1.1 (6)
C8—C9—C10—C11	0.9 (5)	Br23—C19—C20—C21	177.6 (3)
C8—C9—C10—C13	177.7 (4)	C19—C20—C21—C22	1.2 (6)
C9—C10—C11—C12	−0.5 (5)	C20—C21—C22—C17	−2.0 (6)
C13—C10—C11—C12	−177.1 (4)	C18—C17—C22—C21	2.7 (6)
C10—C11—C12—C7	−0.2 (6)	C15—C17—C22—C21	−173.9 (3)
C8—C7—C12—C11	0.3 (5)		

### Hydrogen-bond geometry (Å, °)

**Table:**

*D*—H···*A*	*D*—H	H···*A*	*D*···*A*	*D*—H···*A*
C3—H3···Cg1^i^	0.93	2.85	3.583 (5)	137
C6—H6···Cg1^ii^	0.93	2.78	3.516 (5)	137
C9—H9···Cg2^i^	0.93	2.87	3.544 (5)	131
C12—H12···Cg2^ii^	0.93	2.97	3.655 (5)	131
C21—H21···Cg3^iii^	0.93	2.83	3.505 (5)	131

Symmetry codes: (i) *x*+1/2, −*y*+2, *z*; (ii) *x*−1/2, −*y*+1, *z*; (iii) *x*−1/2, −*y*+2, *z*.
